# Anti-inflammatory Effect of Probiotic *Limosilactobacillus reuteri* KUB-AC5 Against *Salmonella* Infection in a Mouse Colitis Model

**DOI:** 10.3389/fmicb.2021.716761

**Published:** 2021-08-23

**Authors:** Songphon Buddhasiri, Chutikarn Sukjoi, Thattawan Kaewsakhorn, Kowit Nambunmee, Massalin Nakphaichit, Sunee Nitisinprasert, Parameth Thiennimitr

**Affiliations:** ^1^Department of Microbiology, Faculty of Medicine, Chiang Mai University, Chiang Mai, Thailand; ^2^Department of Veterinary Biosciences and Veterinary Public Health, Faculty of Veterinary Medicine, Chiang Mai University, Chiang Mai, Thailand; ^3^Major of Occupational Health and Safety, School of Health Science, Mae Fah Luang University, Chiang Rai, Thailand; ^4^Urban Safety Innovation Research Group, Mae Fah Luang University, Chiang Rai, Thailand; ^5^Department of Biotechnology, Faculty of Agro-Industry, Kasetsart University, Bangkok, Thailand; ^6^Research Center of Microbial Diversity and Sustainable Utilization, Chiang Mai University, Chiang Mai, Thailand; ^7^Faculty of Medicine, Center of Multidisciplinary Technology for Advanced Medicine, Chiang Mai University, Chiang Mai, Thailand

**Keywords:** acute non-typhoidal salmonellosis, *Salmonella enterica* Typhimurium, probiotic *Limosilactobacillus* (*Lactobacillus*), mouse colitis model, anti-inflammatory effect, immunomodulation

## Abstract

Acute non-typhoidal salmonellosis (NTS) caused by *Salmonella enterica* Typhimurium (STM) is among the most prevalent of foodborne diseases. A global rising of antibiotic resistance strains of STM raises an urgent need for alternative methods to control this important pathogen. Major human food animals which harbor STM in their gut are cattle, swine, and poultry. Previous studies showed that the probiotic *Limosilactobacillus* (*Lactobacillus*) *reuteri* KUB-AC5 (AC5) exhibited anti-*Salmonella* activities in chicken by modulating gut microbiota and the immune response. However, the immunobiotic effect of AC5 in a mammalian host is still not known. Here, we investigated the anti-*Salmonella* and anti-inflammatory effects of AC5 on STM infection using a mouse colitis model. Three groups of C57BL/6 mice (prophylactic, therapeutic, and combined) were fed with 10^9^ colony-forming units (cfu) AC5 daily for 7, 4, and 11 days, respectively. Then, the mice were challenged with STM compared to the untreated group. By using a specific primer pair, we found that AC5 can transiently colonize mouse gut (colon, cecum, and ileum). Interestingly, AC5 reduced STM gut proliferation and invasion together with attenuated gut inflammation and systemic dissemination in mice. The decreased STM numbers in mouse gut lumen, gut tissues, and spleen possibly came from longer AC5 feeding duration and/or the combinatorial (direct and indirect inhibitory) effect of AC5 on STM. However, AC5 attenuated inflammation (both in the gut and in the spleen) with no difference between these three approaches. This study demonstrated that AC5 confers both direct and indirect inhibitory effects on STM in the inflamed gut.

## Introduction

Acute gastroenteritis caused by non-typhoidal *Salmonella* is a foodborne disease causing around 93.8 million cases and 155,000 deaths per year worldwide (Majowicz et al., 2010; Heredia and García, 2018; Balasubramanian et al., 2019). The Gram-negative bacterium *Salmonella enterica* is comprised of more than 2,500 serovars. The most important non-typhoidal serovars for human or livestock infections include Typhimurium, Enteritidis, Gallinarum, Infantis, Ohio, Seftenberg, Derby, and Rissen (Antunes et al., 2016; Campos et al., 2019; Ferrari et al., 2019; Sun et al., 2021). The major route of human acute non-typhoidal salmonellosis (NTS) is consumption of contaminated food or drink from animal reservoirs of *Salmonella* (especially cattle, swine, and chickens) (Bernad-Roche et al., 2021). Increased demand for food products in both developed and developing countries leads to the improper use of antibiotics on animal farms. Low dose antibiotics have been used as a growth promoter to increase the yield of meat products (Castanon, 2007). However, this practice results in an increase in multi-drug resistance (MDR) *Salmonella* strains around the world (Zhao and Hu, 2013; Ur Rahman et al., 2018; Sedrakyan et al., 2020). MDR *Salmonella* strains are currently spreading in the human food supply chain and have a huge impact on economics and health (Tasmin et al., 2019; Castro-Vargas et al., 2020; Chen et al., 2020). This concern raised the alarm on the need to find alternative ways to control this important foodborne pathogen (Kongsanan et al., 2020; Mao et al., 2021; Sargun et al., 2021).

*Salmonella enterica* serovar Typhimurium (STM) is a major cause of human acute NTS and its pathogenesis has been extensively investigated (Broz et al., 2012; Rivera-Chavez and Baumler, 2015; Hausmann and Hardt, 2019; Rogers et al., 2021). Once ingested and passed into the large intestine of its mammalian host, STM exploits two type-three secretion systems (T3SS)-1 and T3SS-2 to secrete effector proteins promoting gut epithelial invasion and survival inside host cells, respectively. Innate immune receptors such as Toll-like receptors (TLRs) or nucleotide-binding oligomerization domain-(NOD)-like receptors (NLRs) of gut epithelium recognize pathogen-associated molecular patterns (PAMPs) on STM resulting in gut inflammation (Thiennimitr et al., 2012). Chemotactic cytokines such as interleukin (IL)-8 (CXCL-8 or KC in mouse) and IL-6 promote a neutrophil influx into the gut lamina propria and submucosal tissue at the site of STM entry (Barthel et al., 2003; Tsolis et al., 2011; Santos, 2014). A hallmark histological finding of STM infection in humans is infiltration in numerous polymorphonuclear cells (PMNs) in the large intestine. STM infection also increases the production of inducible nitric oxide synthase (iNOS) derived from gut epithelium and lamina propria inflammatory monocytes (Winter et al., 2013; McLaughlin et al., 2019). Then, iNOS catalyzes the production of nitric oxide (NO), which plays a significant role in the host immune response and *Salmonella* pathogenesis (Lopez et al., 2015). Interestingly, the facultative anaerobic STM can take advantage of gut inflammation to outcompete resident obligate anaerobic gut microbiota and bloom in the inflamed gut (Lupp et al., 2007; Stecher et al., 2007; Barman et al., 2008). In the inflamed gut, alternative terminal electron acceptors such as tetrathionate (S_4_O_6_^2–^) and nitrate (NO_3_^–^) become available for STM. STM exploits these electron acceptors to breakdown non-fermentable molecules such as ethanolamine and 1,2-propanediol by anaerobic respiration (Winter et al., 2010, 2013; Thiennimitr et al., 2011; Faber et al., 2017). This nutritional advantage allows STM to outcompete gut microbiota and flourish in the host gut during the infection (Rivera-Chavez and Baumler, 2015). Successful proliferation in the inflamed gut plays a crucial role in STM transmission to a new host (Stecher et al., 2007; Santos et al., 2009).

Probiotics are defined as “live microorganisms when administered in adequate amounts confer a health benefit on the host” (Morelli and Capurso, 2012). Several probiotics have already been investigated to identify potential beneficial roles against *Salmonella* infection. A Gram-positive bacterium in the genus *Limosilactobacillus* (former named “*Lactobacillus*”) is one of the most common probiotic microorganisms used to promote animal and human health (Kakabadze et al., 2020; Petrova et al., 2021). Several species of *Limosilactobacillus*, for example, *reuteri*, *paracasei*, *johnsonii*, *plantarum*, and *rhamnosus* are categorized as generalized recognized as safe (GRAS) and can be used as probiotics. Previous studies have already demonstrated an anti-*Salmonella* effect of the probiotic *Limosilactobacillus* both *in vitro* and *in vivo* (Nakphaichit et al., 2019; Hai et al., 2021; Jia et al., 2021; Kim et al., 2021). Effects of probiotics against enteropathogens (colonization resistance) can be divided into two major branches: (1) direct effect including prevention of pathogen adhesion and invasion to gut epithelium, nutritional competition with pathogens, or direct killing activity on pathogens by the production of antimicrobial substances and (2) indirect effect by enhancing host protective immune response (locally or systemically) such as strengthening gut barrier integrity, increasing production of secretory IgA (SIgA) antibody or attenuation of inflammatory responses (Sassone-Corsi and Raffatellu, 2015). Several strains of *Limosilactobacillus* reduced *Salmonella* lipopolysaccharide (LPS)-induced gut epithelial barrier impairment by increasing tight junction protein expressions (Fang et al., 2010; Yeung et al., 2013). *Limosilactobacillus* probiotics attenuated *Salmonella*-induced gut inflammation by reducing pro-inflammatory cytokine production by the attenuation of TLRs activation *in vitro* (Kanmani and Kim, 2020). Kanmani and Kim (2020) showed that *Limosilactobacillus* isolated from Korean food increased production of the immunosuppressive cytokine IL-10, transforming growth factor (TGF)-β, and antimicrobial peptide β-defensin by manipulating the expression of TLR negative regulators in human colonocytes.

Liu et al. (2019) demonstrated anti-*Salmonella* activity of *Limosilactobacillus* in mice. They found that *L. plantarum* ZS2058 and *L. rhamnosus* GG (LGG) reduced *Salmonella* pathogenicity and inflammatory response in a mouse typhoid model with a strain-specific mechanism. The probiotic *L. plantarum* ZS2058 increased fecal short chain fatty acid (propionic acid) and mucin levels in mouse colon, while LGG more strongly alleviated mouse gut inflammation. This study reported the anti-*Salmonella* activity of the probiotic *Limosilactobacillus* in mice. However, mice infected with STM do not properly develop a robust gut inflammation but rather have a systemic infection called “typhoid fever-like disease” (Tsolis et al., 1999, 2011). To study the anti-inflammatory role of the probiotic *Limosilactobacillus* against an acute NTS, a mouse colitis model for *Salmonella* infection should be used. Antibiotic pretreatment in the genetically susceptible mouse strains (e.g., C57BL/6 and BALB/c) prior to STM infection transiently abrogates mouse colonization resistance. Then, STM triggers mouse gut inflammation which mimics acute gastroenteritis in human (Bohnhoff et al., 1954; Bohnhoff and Miller, 1962).

The anti-*Salmonella* effect of the probiotic AC5 has already been investigated in broiler chickens (Nakphaichit et al., 2011, 2019; Sobanbua et al., 2019). Nakphaichit et al. (2019) recently reported that oral feeding with AC5 increases the survival rate and attenuates the pathogenicity of *Salmonella* infection in chickens by modulating chicken gut microbiota and immune responses. Due to the differences in the gut immune response between avian and mammalian hosts when interacting with STM, the outcomes of probiotic *Limosilactobacillus* challenge between these two hosts might differ (Tsolis et al., 1999; Santos et al., 2001). Here, we investigated the anti-*Salmonella* and anti-inflammatory effects of the probiotic *L. reuteri* KUB-AC5 in the inflamed gut of STM-infected mice.

## Materials and Methods

### Ethical Approval

The animal experiments in this study were approved by the Animal Care and Use Committee, Chiang Mai University, Thailand in accordance with the Association for Assessment and Accreditation of Laboratory Animal Care (AAALAC) guidelines (Approval No. 2559/MC-0005).

### Bacterial Strains, Culture Conditions, and Direct Antimicrobial Effect Assays

The *S.* Typhimurium strain IR715 (STM) is a fully virulent, nalidixic acid-resistant derivative of the wild-type isolate ATCC 14028 (Stojiljkovic et al., 1995). The probiotic *L. reuteri* KUB-AC5 (AC5) was isolated from chicken intestine (Nitisinprasert et al., 2000). To determine the direct anti-*Salmonella* activity of AC5, we performed growth assays in both liquid (co-culture) and solid media (spot-on lawn and agar-well diffusion assays). For the co-culture assay, 100 μL of 1:100 dilution of an equal amount (1:1) of STM and AC5 inocula [about 10^4^ colony-forming unit (cfu)/mL total] was incubated microaerobically (without shaking) in 10 mL of co-culture broth at 37°C for 16 h as previously described with a slight modification (Drago et al., 1997; Adetoye et al., 2018). The co-culture broth was composed of an equal amount of the double strength (2×) de Man, Rogosa, and Sharpe (MRS) and Mueller–Hinton (MH) broth. For a single growth assay, 100 μL of 1:100 dilution of an overnight culture of AC5 or STM was inoculated separately into 10 mL fresh MRS and MH broth, respectively, and then incubated at 37°C for 14 h without shaking. At the indicated time point, viable counts (cfu/mL) of AC5 and STM were enumerated using a serial 10-fold dilution with the appropriate media MRS agar (AppliChem) and Luria–Bertani (LB) agar with 0.05 mg/mL nalidixic acid, respectively.

The direct anti-*Salmonella* effect of the viable cell and cell-free supernatant from AC5 culture (AC5-CFS) of probiotic AC5 was determined by spot-on lawn and agar well diffusion methods as described previously (Lima et al., 2007). For the spot-on lawn assay, 20 μL of AC5 overnight culture was spotted on MRS agar and incubated at 37°C for 16 h to develop a viable spot on the plate. Then, 200 μL of STM overnight culture was added to 20 mL LB broth containing 0.75% agar (Difco agar, BD, MD, United States) and poured over the plate. All plates were incubated at 37°C for 16 h to observe the inhibition zone. A diameter of the zone of inhibition more than 1 mm was considered as a positive.

An agar well diffusion method was used to evaluate the antimicrobial effect of AC5-CFS on STM as previously described (Lima et al., 2007). To collect AC5-CFS, 5 mL of AC5 overnight culture was centrifuged at 4,000 rounds per minute (rpm) for 10 min at 4°C. An STM lawn was prepared by mixing 200 μL of STM overnight culture in 20 mL LB with 0.75% agar and then pouring into a sterile plate. Then, 6-mm-diameter wells were made in the agar by 200 μL pipette tip and filled with 60 μL of AC5-CFS. All plates were incubated at 37°C for 16 h to observe the inhibition zone. The inhibition activity was expressed as a clear zone.

### Probiotic *L*. *reuteri* KUB-AC5 Preparation

A single fresh colony of the probiotic *L. reuteri* KUB-AC5 grew on MRS agar (Difco agar, BD, MD, United States) with 0.6% w/v CaCO_3_ was picked and inoculated into 5 mL MRS broth with 0.6% w/v CaCO_3_. The mixture was statically incubated at 37°C for 16–18 h. Then, 400 μL AC5 overnight culture was added into a new 40 mL MRS broth with 0.6% w/v CaCO_3_ (1:100 dilution) and grown statically for the next 16–18 h. The bacterial culture was centrifuged at 4,000 rpm at 4°C for 10 min to obtain the cell pellets which were resuspended with sterile PBS to adjust the final concentration of 10^10^ cfu/mL and kept at 4°C until use.

### Animal Study

The animal experiments were approved by the Animal Care and Use Committee, Chiang Mai University, Thailand in accordance with the AAALAC guidelines (Approval No. 2559/MC-0005). Female C57BL/6 mice aged 6–8 weeks old were purchased from Nomura Siam International (Bangkok, Thailand). All mice were acclimatized and co-housed (for gut microbiota normalization) for 1 week and housed in 12 dark/12 light hour cycle in a controlled environment (room temperature, 21 ± 1°C; humidity, 50 ± 10%). Standard mouse chow and drinking water were provided *ad libitum*. Then, mice were divided into four groups: (1) prophylactic (Pro), (2) therapeutic (Tx), (3) combined (prophylactic and therapeutic, Pro + Tx), and (4) untreated control group. There were six to seven mice per group. Mouse was orally fed with 100 μL of 10^10^ cfu/mL AC5 (10^9^ cfu/mouse) daily for 7, 4, and 11 days for the prophylactic, therapeutic, and combined group, respectively. Mice in the untreated control group were daily fed with sterile PBS. To induce colitis, 100 μL of (200 mg/mL) streptomycin sulfate (Sigma Aldrich, Singapore) solution was given orally to mice 1 day prior to STM infection (10^9^ cfu of STM/mouse) (Barthel et al., 2003; Sarichai et al., 2020). All mice were euthanized at day 4 post infection.

### Determination of Bacterial Numbers

A viable count (cfu/gm tissue) of STM in mouse gut contents (colon content and cecal content), gut tissues (colon, cecum, and ileum), and spleen was performed using a serial-dilution plating technique. Briefly, the samples were collected in sterile PBS and homogenized with a bead-beating machine with 1.0 mm diameter zirconium/silica beads (Biospec Products, Bartlesville, OK, United States). Then, serial 10-fold dilutions of the homogenates were made. STM cfu/gm tissues were enumerated by plating 100 μL of the dilutions onto LB agar with nalidixic acid (0.05 mg/mL) and incubated at 37°C for 16 h.

*Limosilactobacillus reuteri* KUB-AC5 was quantified by quantitative polymerase chain reaction (qPCR) with the AC5 strain-specific primer pair (Sobanbua et al., 2019). The forward and reverse primers were TCGCTCACGGCTGTTAGGACA and AGCACTCCACGTTGCCACA, respectively. The microbial genomic DNA was extracted from mouse gut contents by using the QIAamp Stool Mini Kit (Qiagen, Hilden, Germany) in a combination with the bead beating method. About 100–200 mg of gut contents was suspended in 900 μL of sterile PBS (pH 8.0), 300 μL phenol-chloroform-isoamyl alcohol (25:24:1) and 0.3 g of zirconium beads (0.1 mm in diameter, As One Corporation, Osaka, Japan). Microbial cells in gut contents were broken by a minibead beater 3110BX (Biospec, United States) for 3 min at 4,800 rpm and further DNA extraction was done in accordance with the protocol of the QIAamp Stool Mini Kit. Total bacterial DNA was applied as a template for qPCR. Total qPCR reaction volume of 20 μL contained 50 ng/μL of DNA template, 2 μL; 10 μmol of each forward and reverse primer, 0.4 μL; LightCycler 480 SYBR Green master (Roche, Germany), 10 μL; and nuclease-free water added to obtain the final volume of 20 μL. Amplification conditions of initial denaturation at 95°C for 5 min were followed by 45 cycles of 95°C for 10 s, annealing and extension step combined at 70°C for 45 s, and elongation at 72°C for 5 min. A serial 10-fold dilution of genomic DNA of *L. reuteri* KUB-AC5 was used to generate the standard curve.

### Detection of Tissue Gene Expression by qPCR

Four days after the infection with STM, all mice were euthanized and tissue samples were collected for mRNA expressions by qPCR. Fold change (relative of expression) of important proinflammatory cytokine genes [*Kc*, *Ifng*, and *Il-6* encoding for KC or CXCL-1, interferon (IFN)-gamma and IL-6, respectively], *Nos2* and *Zo-1* [encoding for iNOS and a tight junction protein zonula occludens (ZO-1), respectively], were performed as previously described (Sarichai et al., 2020). In short, collected tissues were immersed in RNA preservative solution (RNAstore reagent, TIANGEN, China) and stored at −20°C until extraction. Mouse tissue RNA was isolated using TRIzol reagent (Thermo Fisher scientific, Waltham, MA, United States), following the manufacturer’s protocol. DNase treatment was performed using a DNA removal and inactivation kit (Ambion, Life technologies, Vilnius, Lithuania). The purity and amount of the isolated RNA were confirmed using a NanoDrop Spectrophotometer (Thermo Fisher scientific, Waltham, MA, United States). Then, complimentary DNA (cDNA) was synthesized using a Tetro cDNA synthesis kit (Bioline, Taunton, MA, United States). The qPCR reaction was prepared by using a SensiFAST SYBR Lo-ROX Kit (Bioline, Taunton, MA, United States) and performed with ViiA 7 Real-Time PCR system (Applied Biosystems). Gene expressions were analyzed with a comparative *Ct* method (2^–Δ^ ^Δ^ ^*Ct*^) using a *Gapdh* as a housekeeping genes (Schmittgen and Livak, 2008). The lists of primers used in this study are shown in Supplementary Table 1.

### Cecal Histopathological Study

Segments of mouse ceca were fixed in 10% buffered formalin, embedded in paraffin, and stained with hematoxylin and eosin (H&E). The slides were blindly scored by the veterinary pathologist using the criteria shown in Supplementary Table 2.

### Statistical Analysis

The cfu/gm of STM and relative fold changes of mRNA were logarithmically transformed before statistical analysis. Analysis was carried out using a parametric test [one-way analysis of variance (ANOVA) with Tukey’s multiple comparisons test]. The cecal histopathological scores were statistically tested using a Kruskal–Wallis test with multiple comparisons as previously described (Sarichai et al., 2020). *P* < 0.05 was considered statistically significant; ^∗^, ^∗∗^, and ^∗∗∗^ indicate *P*-values < 0.05, 0.01, and 0.001, respectively.

## Results

### *Limosilactobacillus reuteri* KUB-AC5 Exhibited Direct Antimicrobial Activity on *Salmonella* Typhimurium

**FIGURE 1:**
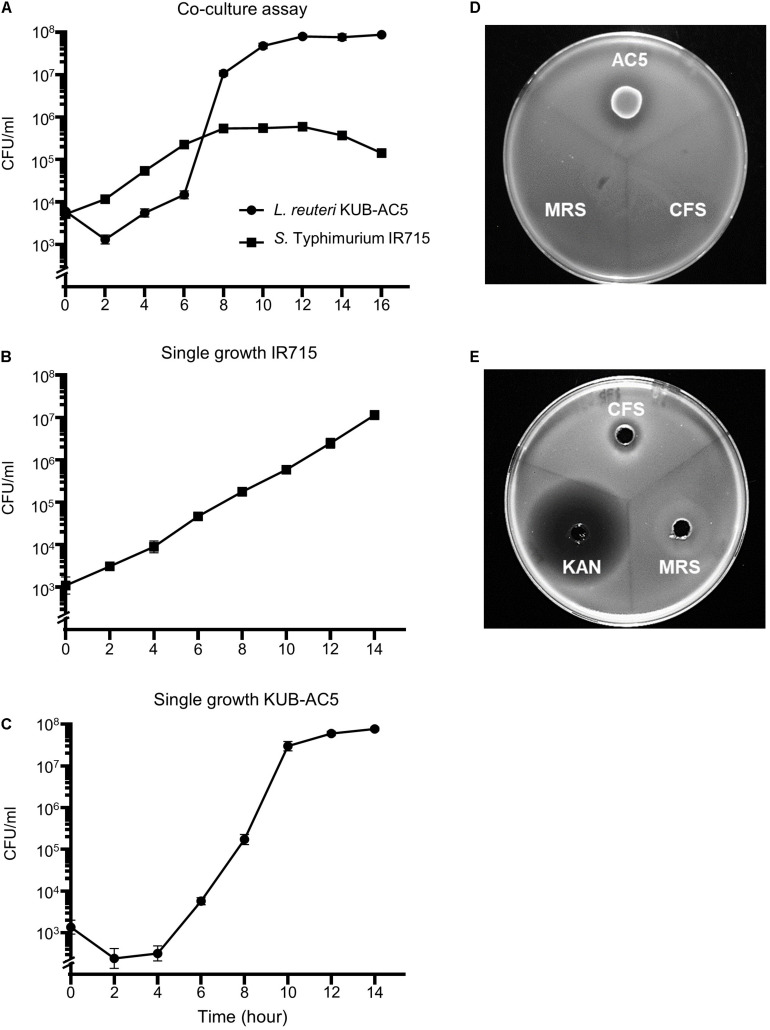
*In vitro* anti-*Salmonella* effect of probiotic *L. reuteri* KUB-AC5 on *S.* Typhimurium IR715 in liquid and solid media. Co-culture assay was done by inoculating an equal amount of AC5 and STM in de Man, Rogosa, and Sharpe (MRS) and Mueller–Hinton (MH) broth **(A)**. An independent growth of STM and AC5 in liquid media was performed (**B,C**, respectively). The anti-*Salmonella* activities of AC5 cell-free supernatant (CFS) were tested using spot-on lawn assay **(D)** and agar well diffusion assay **(E)**. Data represent geometric means ± SD of three independent experiments. KAN, kanamycin as a positive control, MRS broth as a negative control.

In a previous study, the strain AC5 produced antimicrobial peptide KAC5 of 4.7 kD against *S.* Enteritidis S003 with bactericidal activity in its mode of action (Sobanbua et al., 2020). To investigate whether the AC5 can inhibit growth of STM IR715 used in this study, the time course growth assays of both monoculture and co-culture were carried out as shown in Figure 1. Co-culture assay demonstrated that probiotic AC5 can inhibit the growth of STM and maintain at 10^5^ cfu/mL (Figure 1A) during 8–14 h compared to that of 10^7^ cfu/mL in the monoculture assay (Figure 1B). From the spot-on lawn assay, we found the clear zone only around the viable colonies of AC5 but not CFS (Figure 1D). By the agar well diffusion assay, we observed the clear zone around the wells of both CFS and KAN (positive control) at 16 h after the inoculation but not the MRS (negative control) (Figure 1E). The diameters of clear zone in both assays are depicted in Supplementary Figure 2. These data indicated the direct antagonistic effect of both viable cells and CFS of AC5 on STM IR715.

### Oral Feeding With *L*. *reuteri* KUB-AC5 Reduced Numbers of *Salmonella* in Mouse Inflamed Gut

**FIGURE 2:**
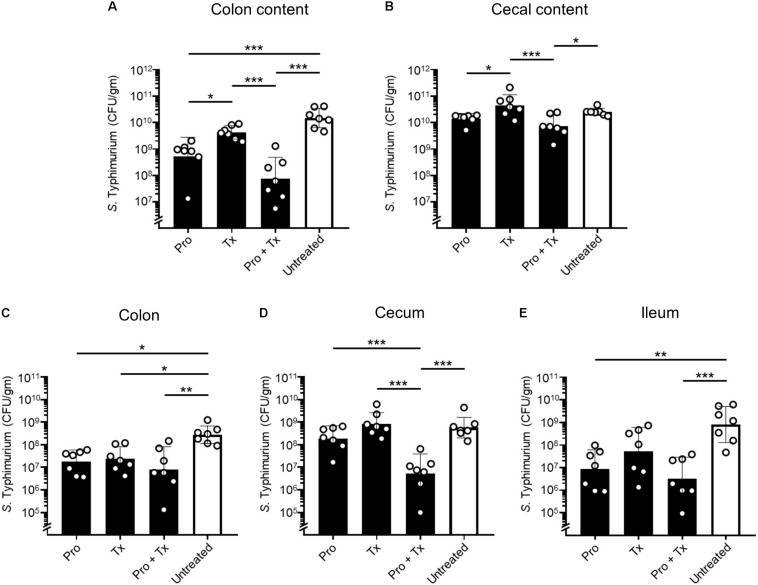
Anti-*Salmonella* effect of probiotic *L. reuteri* KUB-AC5 in mouse inflamed gut. Female C57BL/6 mice were fed daily with 10^9^ cfu AC5 in prophylactic (Pro), therapeutic (Tx), and combined (Pro + Tx) approaches for 7, 4, and 11 days, respectively (black bar). Mice in the untreated control group were fed with phosphate-buffered saline (PBS) (white bar). Then, mouse tissues were harvested at day 4 post infection and homogenized for the enumeration of STM cfu/gm tissues. Bars represent geometric means ± SD. *P < 0.05, **P < 0.01, and ***P < 0.001 compared to all other groups.

To study the role of AC5 consumption in an acute NTS, a mouse colitis model of STM infection was used. The 6–8-week-old female C57BL/6 mice were divided into four groups: (1) prophylactic (Pro), (2) therapeutic (Tx), (3) combined (prophylactic and therapeutic, Pro + Tx), and (4) untreated group. Mice in the treatment groups were orally fed daily with 10^9^ cfu AC5 at the different indicated times (Supplementary Figure 1). At day 4 post infection, all mice were euthanized and tissue samples were harvested. Our data indicated that all groups of mice fed with AC5 have decreased numbers of STM in their gut contents (colon content and cecal content) (Figures 2A,B). Mice in the combined group (Pro + Tx) showed a remarkable decrease in STM numbers in their colon contents. To evaluate the role of AC5 against STM gut tissue invasion, STM cfu/gm from different sites of the gut (colon, cecum, and ileum) were determined. Our data showed that all AC5-fed mice had significantly lower STM numbers in their gut tissues than those of the untreated mice (Figures 2C–E).

### The Probiotic *L*. *reuteri* KUB-AC5 Transiently Colonized Mouse Gut

**FIGURE 3:**
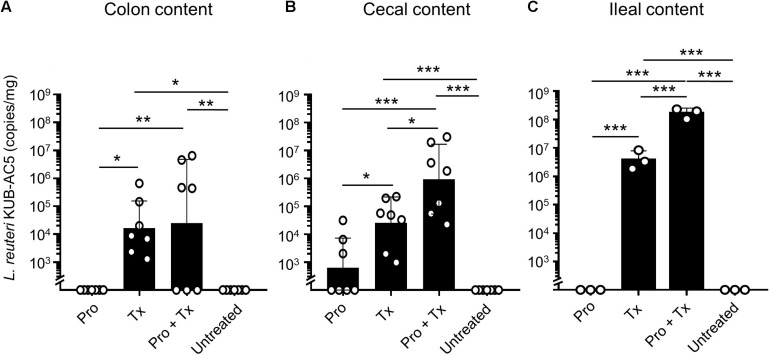
Detectable amounts of *L. reuteri* KUB-AC5 in mouse gut contents. At day 4 post infection, mouse gut contents in colon, cecum, and ileum were collected and quantified for AC5 by qPCR. The copies/mg of AC5 in each gut content was calculated and depicted. Bars represent geometric means ± SD. *P < 0.05, **P < 0.01, and ***P < 0.001 compared to all other groups.

To detect the presence of AC5 in mouse gut, we used a qPCR technique with a primer pair specific to AC5. Then, copy numbers of AC5 per mg of mouse gut content were calculated as previously described (Sobanbua et al., 2019). Our data demonstrated that AC5 can colonize differently in mouse colon, cecum, and ileum (Figure 3). Interestingly, the difference in AC5 feeding duration might play a role in a significant difference of AC5 amounts collected from mouse gut. In the therapeutic (Tx) and combined (Pro + Tx) groups, mice were orally fed with AC5 continuously for 4 and 11 days, respectively, before the sample collection. At the same time, mice in the prophylactic (Pro) group were fed with AC5 for 7 days and stopped for 4 days before the sample collection. Our results showed that mice in the prophylactic group have the lowest amount of AC5 in their gut contents. These data suggest the transient manner of AC5 colonization in mouse gut.

### The Probiotic *L*. *reuteri* KUB-AC5 Attenuated Gut Inflammation During *Salmonella* Infection

**FIGURE 4:**
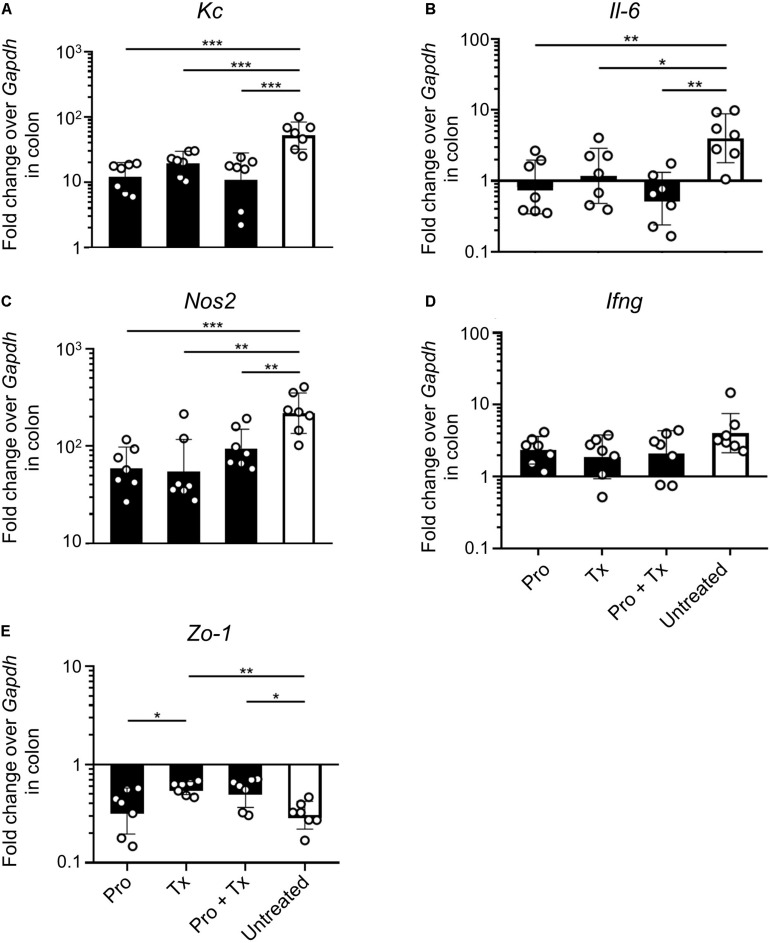
Anti-inflammatory effect of *L. reuteri* KUB-AC5 on the STM-infected mouse colon. At day 4 post infection, mouse colons were collected and quantified for the fold change in mRNA expression of pro-inflammatory cytokines (*Kc*, *Il-6*, and *Ifng*), iNOS (*Nos2*), and tight junction protein (*Zo-1*) by qPCR. All genes were analyzed with the comparative *Ct* method over the housekeeping gene *Gapdh*. Bars represent geometric means ± SD. *P < 0.05, **P < 0.01, and ***P < 0.001 compared to all other groups.

Next, we investigated the anti-inflammatory effect of AC5 on mouse gut. Relative expressions of the representative pro-inflammatory genes were determined by qPCR. Our results revealed that all AC5-fed mice have reduced expression levels of colonic pro-inflammatory genes (*Kc*, *Il-6*, and *Nos2*) (Figures 4A–C, respectively). However, the colonic gene expressions of *Ifng* encoding for cytokine IFN-γ, an important cytokine in the systemic phase of STM infection, were not changed in all groups (Figure 4D). Therapeutic (Tx) and combined (Pro + Tx) orally fed with the probiotic AC5 mice showed increased expression of tight junction protein zonula occludens gene (*Zo-1*) in the colon (Figure 4E).

**FIGURE 5:**
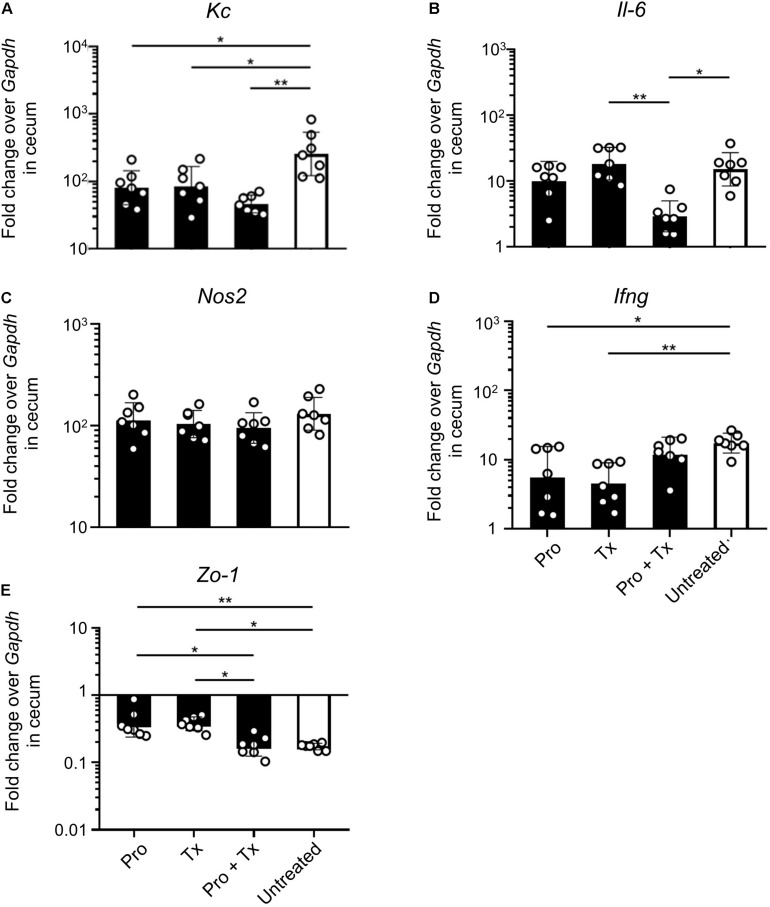
Anti-inflammatory effect of *L. reuteri* KUB-AC5 on the STM-infected mouse cecum. At day 4 post infection, mouse ceca were collected and quantified for the fold change in mRNA expressions of pro-inflammatory cytokines (*Kc*, *Il-6*, and *Ifng*), iNOS (*Nos2*), and tight junction protein (*Zo-1*) by qPCR. All genes were analyzed with the comparative *Ct* method over the housekeeping gene *Gapdh*. Bars represent geometric means ± SD. *P < 0.05 and **P < 0.01 compared to all other groups.

**FIGURE 6:**
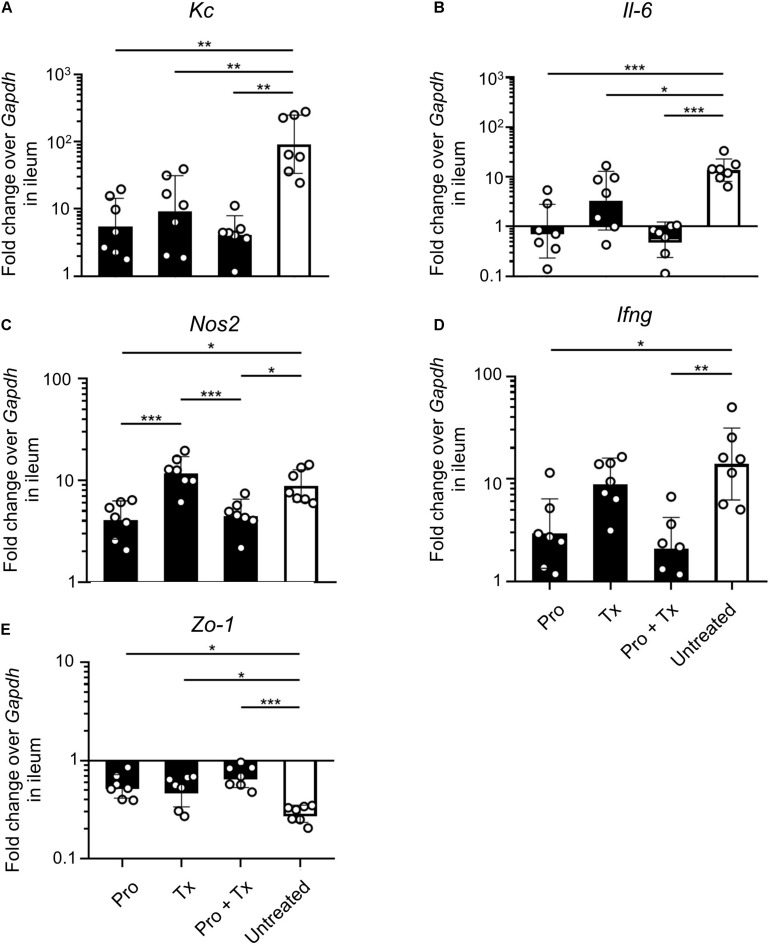
Anti-inflammatory effect of *L. reuteri* KUB-AC5 on the STM-infected mouse ileum. At day 4 post infection, mouse ilea were collected and quantified for the fold change in mRNA expressions of pro-inflammatory cytokines (*Kc*, *Il-6*, and *Ifng*), iNOS (*Nos2*), and tight junction protein (*Zo-1*) by qPCR. All genes were analyzed with the comparative *Ct* method over the housekeeping gene *Gapdh*. Bars represent geometric means ± SD. *P < 0.05, **P < 0.01, and ***P < 0.001 compared to all other groups.

**FIGURE 7:**
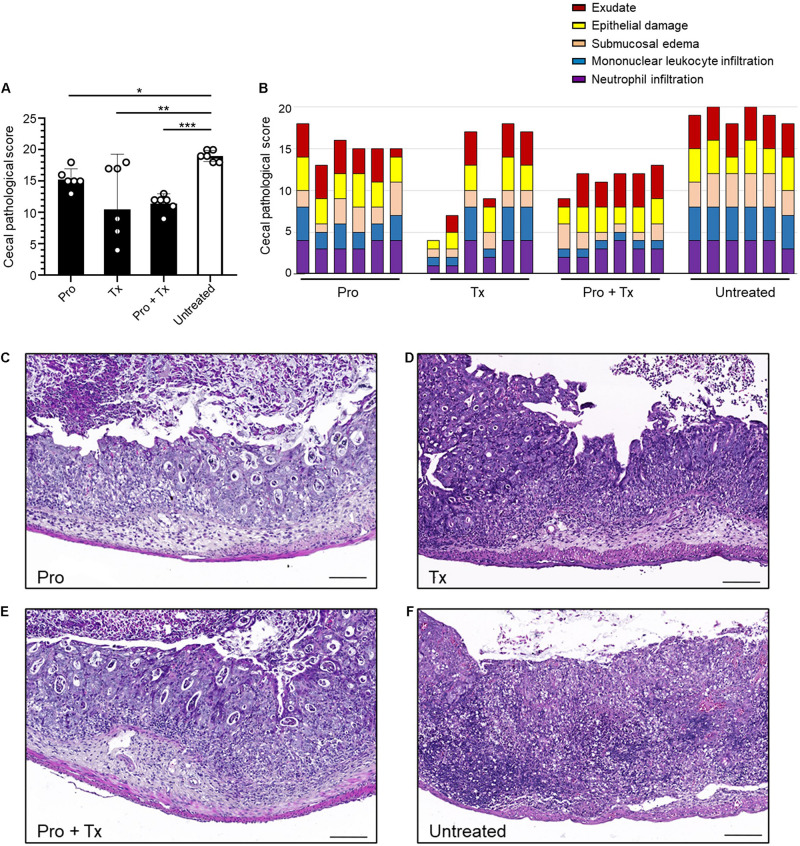
Decreased cecal histopathological scores in STM-infected mice orally fed with *L. reuteri* KUB-AC5. At day 4 post infection, mouse ceca were sectioned and stained for hematoxylin and eosin to evaluate the severity of gut inflammation. Exudate, epithelial damage, submucosal edema, mononuclear leukocyte infiltration, and neutrophil infiltration were assessed. The graphs display total pathological score **(A)** and individual score **(B)**. The representative pictures of prophylactic (Pro), therapeutic (Tx), combined (Pro + Tx), and untreated **(D)** are shown in **(C–F)**, respectively. Bars represent geometric means. *P < 0.05, **P < 0.01, and ***P < 0.001 compared to all other groups.

STM-infected mice orally fed with AC5 also showed reduced gene expressions of *Kc* and *Il-6* in their ceca (Figures 5A,B). There was no significant change in cecal *Nos2* expression in all groups of mice (Figure 5C). Mice in the prophylactic (Pro) and therapeutic (Tx) groups showed a reduction in *Ifng* gene expression (Figure 5D). *Zo-1* was upregulated in the prophylactic and therapeutic groups (Figure 5E). An anti-inflammatory effect of AC5 in the distal ileum of STM-infected mice was also observed (Figure 6). Probiotic AC5 feeding decreased *Kc*, *Il6*, *Nos2*, *Ifng*, and increased *Zo-1* expressions in the ilea of the mice. To confirm the anti-inflammatory effect of AC5 on gut inflammation, tips of mouse ceca were sectioned, stained, and evaluated. The histological study revealed that AC5 feeding attenuated the severity of gut inflammation induced by STM compared to that of the untreated mice (Figure 7).

### The Probiotic *L*. *reuteri* KUB-AC5 Reduced Systemic Dissemination of *Salmonella* Typhimurium in Mice

**FIGURE 8:**
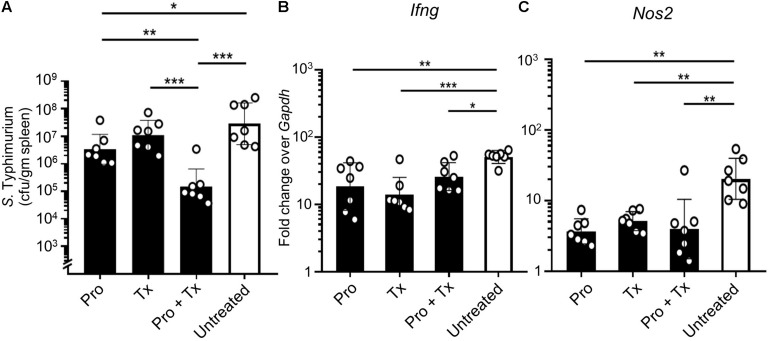
Probiotic *L. reuteri* KUB-AC5 attenuates a systemic dissemination of STM. AC5-fed mice were divided into three groups (Pro, Tx, and Pro + Tx, black bar) and one untreated group (white bar). Mouse spleens were harvested at day 4 post infection and homogenized for STM cfu/gm determination **(A)**. Fold change of *Ifng* and *Nos2* genes in mouse spleen was calculated by qPCR (**B,C**, respectively). All genes were analyzed with the comparative *Ct* method over the housekeeping gene *Gapdh*. Bars represent geometric means ± SD. *P < 0.05, **P < 0.01, and ***P < 0.001 compared to all other groups.

In the systemic phase of infection, STM survives inside the cytoplasmic vacuoles of host innate immune cells such as macrophages or monocytes. This intracellular niche protects STM from host immune recognition and allows STM to disseminate through lymphatic and blood vessels to distant organs such as the spleen (Broz and Monack, 2011; Broz et al., 2012). Our data showed that feeding mice with AC5 significantly reduced STM numbers in the spleen (Figure 8A). The combined regimen (Pro + Tx) of AC5 provided the greatest effect in the reduction of the STM numbers in the mouse spleen. AC5 also decreased the expressions of *Ifng* and *Nos2* in mouse spleen (Figures 8B,C). These data indicated that orally fed with probiotic *L. reuteri* KUB-AC5 can reduce the severity the systemic phase of STM infection in mice.

## Discussion

STM is one of the most prevalent foodborne pathogens causing several million cases of acute gastroenteritis and invasive bacteremia in humans per year (Majowicz et al., 2010; Whistler et al., 2018). Although the most outcome of STM infection is self-limiting acute diarrhea, a life-threatening condition could occur in immunocompromised hosts such as those with human immunodeficiency virus (HIV)-infection, malnutrition, or extreme ages (Gordon et al., 2002; Preziosi et al., 2012). An increase in MDR STM necessitates an urgent alternative approach for this important foodborne pathogen. STM is a frank pathogen which can be recognized by host gut immune receptors and exploit host responses to its benefit (Baumler et al., 2011; Rivera-Chavez and Baumler, 2015). By using its virulence factors, a minor population of STM can invade and survive inside host cells while the majority proliferates in the gut lumen of the host (Santos et al., 2009). Winter et al. (2010) showed that STM conferred a nutritional advantage from gut inflammation, outcompeted resident gut microbiota, and then bloomed in the inflamed gut. Gut inflammation provides several host-derived resources for STM to flourish in the gut. For example, increased oxygen, nitrate, tetrathionate, and lactate levels in the inflamed gut have been reported (Winter et al., 2010; Lopez et al., 2012; Faber et al., 2017; Rogers et al., 2021). These evidences suggest that the facultative anaerobic bacteria such as STM can harness the host response (gut inflammation) to outcompete the indigenous gut microbiota in the inflamed gut. Hence, strategies that reduce gut inflammation might also decrease STM blooming in the host intestinal tract.

Probiotic microorganisms are a promising way to be used as an alternative to antibiotics in several infectious diseases including an acute NTS (Storr and Stengel, 2021). Two major mechanisms used by probiotics in the inhibition of STM growth have been reviewed (Sassone-Corsi and Raffatellu, 2015). (I) Probiotics could directly inhibit growth of STM by adhesion exclusion, competing for nutrient sources of antimicrobial peptide production. (II) Some probiotics confer an indirect (immunomodulation) effect on gut immunity such as increasing gut barrier integrity and enhancing production of anti-inflammatory cytokines or protective SIgA antibody against STM. The anti-*Salmonella* effect of probiotic *L. reuteri* KUB-AC5 has been recently reported (Nakphaichit et al., 2019). Nakphaichit et al. (2019) showed that high dose AC5 consumption for 3 days prevents *Salmonella* infection in chickens. The gut microbiota of chickens supplemented with AC5 was altered by causing an increase in *Lactobacillaceae* and a decrease in *Enterobacteriaceae* in their ilea and ceca. These results indicate the gut microbiota modulation effect of AC5 on *Salmonella* infection in chickens. Although the anti-inflammatory effect of probiotic *Limosilactobacillus* on *Salmonella* in other experimental models has already been investigated (Abhisingha et al., 2018; Mohanty et al., 2019; Shi et al., 2019; Smialek et al., 2019; Acurcio et al., 2020; Kanmani and Kim, 2020; Kowalska et al., 2020; Mizuno et al., 2020), the strain-specific effect of the probiotic plays a crucial role in their different outcomes.

Liu et al. (2018) showed that prophylactic oral feeding with *L. plantarum* and *L. rhamnosus* reduced *Salmonella* numbers and improved gut barrier integrity in C57BL/6 mice. Reduced translocating *Salmonella* numbers were found in the spleen and liver of *L. rhamnosus* HN001 orally fed BALB/c mice (Gill et al., 2001). Interestingly, *L. casei* CRL431 enhanced the production of IFN-γ levels in immune cells isolated from Peyer’s patch of mice infected with STM. Castillo et al. (2011) found that the combined effect (Pro + Tx) resulted in increased IFN-γ and IL-6 levels in mouse intestinal fluid. Nonetheless, most of the previous studies into the probiotic effect of *Limosilactobacillus* against STM were performed in a non-robust gut inflammation mouse typhoid model. Here, we used a streptomycin-pretreated mouse colitis model to investigate anti-*Salmonella* and anti-inflammatory effects of *L. reuteri* KUB-AC5.

The direct anti-*Salmonella* effect of AC5 is shown in Figure 1. We investigated the direct antagonistic effects of AC5 on STM using co-culture growth, spot-on lawn, and agar well-diffusion assays. Our data showed that the direct antagonism of AC5 on STM could arise from either the cells or cell-free components of AC5. These data confirmed the anti-*Salmonella* activity of AC5 *in vitro* as previously shown (Nitisinprasert et al., 2000). Previous studies also demonstrated that AC5 inhibited *in vitro* growth of several strains of *Salmonella* and *Escherichia coli* by production of short chain fatty acids and antimicrobial peptides not as a result of the acidity effect. Recently, the antimicrobial peptide KAC5 produced by AC5 was characterized and was shown to have no similarity to bacteriocin (Sobanbua et al., 2020). KAC5 of AC5 demonstrated a broad inhibition spectrum against several Gram-positive and -negative bacteria but not against lactic acid-producing bacteria. Next, we compared the role of prophylactic, therapeutic, and combined (prophylactic and therapeutic) AC5 feeding on STM infection in mice. Our data showed that all groups of AC5-fed mice had decreased STM numbers in several parts of their gut and spleen. Interestingly, AC5 caused a more significant reduced in STM numbers in the colon content than in the cecal content in all groups of treated mice. This suggests that the antagonistic activity of AC5 is gut site-specific on STM in mice. A previous study by Jiang et al. (2019) showed that intraperitoneal administration of *L. reuteri* ATCC 55730 (both live and heat-inactivated forms) reduced severity of an invasive NTS in mice by macrophage activation. In this study, we demonstrated the role of oral consumption of *L. reuteri* KUB AC-5 in both prevention and treatment of an acute NTS. The combined feeding (Pro + Tx) of AC5 resulted in the highest anti-*Salmonella* effect in the mouse gut lumen (gut contents) compared to the other feeding approaches. These data suggest a positive role of AC5 feeding and its duration on the inhibition of STM proliferation and invasion in the gut of mice.

Previous studies revealed that AC5 predominated in the ileum and cecum of chicken (Nakphaichit et al., 2011; Sobanbua et al., 2019). Interestingly, our data showed that AC5 could be detected in mouse colon, cecum, and ileum with the highest numbers in the ileum (Figure 3). Mice in the combined group also had the highest amount of AC5 compared to that of the therapeutic and prophylactic groups. Mice in the prophylactic group had the lowest amount of AC5 in their colon, cecum, and ileum. These results indicated that AC5 can transiently colonize the gut of mice and is dependent on feeding duration.

Next, we investigated the anti-inflammatory activity of AC5 on STM infection. Non-typhoidal *Salmonella* induces a robust gut inflammation in several agricultural animal hosts including cattle, swine, and poultry (Fasina et al., 2008; Bai et al., 2014; Yu et al., 2017; Dar et al., 2019; Huang et al., 2019). However, to date there are few reports on the *in vivo* gut inflammatory attenuating effect of probiotic *Limosilactobacillus* (Yu et al., 2017; Pradhan et al., 2019; Peng et al., 2020). Our data showed that AC5 reduced the gene expression of pro-inflammatory cytokine (*Kc*, *Il-6*, and *Ifng*) and *Nos2* in mouse colon, cecum, and ileum. The tight junction protein gene (*Zo-1*) expression was upregulated in the gut of AC5-fed mice (Figures 4–7). However, different gene expressions were observed in different sites of mouse gut. The results from a prophylactic group of mice (fed with AC5 for 7 days before STM infection) could indicate, at least in part, an indirect inhibitory growth effect of AC5 on STM (Supplementary Figure 1). Our data show that numbers of STM in the gut lumen of the prophylactic group were reduced in comparison to that of the therapeutic group (Figures 2A,B), while AC5 can transiently colonize mouse gut (Figure 3). However, the degree of gut inflammation is also depending on the numbers of STM in the gut lumen, and vice versa (Winter et al., 2010). By enhancing the nutrient advantage for facultative anaerobes in the inflamed gut lumen, the reduced gut inflammation could come from a direct inhibitory effect of AC5 on STM in the gut lumen as well. Surprisingly, there was no statistically significant difference in the attenuation of gut inflammation between groups of AC5-fed mice. Reduced gut inflammation by AC5 feeding might be due to the decrease in STM proliferation by limiting their necessary resources such as host-derived nutrients. In addition, AC5 prevented the systemic dissemination of STM from the gut into spleen in this model perhaps due to the luminal STM population in the gut (Figure 8). The lowest disseminated numbers of STM in spleen were found in the combined group. This suggested that the beneficial role of AC5 in an invasive NTS may come from either the duration of feeding of AC5 or an additional effect of direct and indirect colonization resistance of AC5. However, a mouse typhoid model (without antibiotic pretreatment) should be used for further investigation into whether AC5 attenuates the severity of systemic disease caused by *Salmonella*.

## Conclusion

Oral administration of the probiotic *L. reuteri* KUB-AC5 reduced the severity of acute NTS in a mouse model. *In vivo* anti-*Salmonella* (decreasing numbers of STM) and anti-inflammatory (attenuation of gut and spleen inflammation) effects of AC5 have been revealed. AC5 is more effective against STM when given as a combination (prophylactic and therapeutic) indicating the roles of feeding duration together with the combinatorial effects (direct and indirect colonization resistance) on the probiotic activities of AC5. However, other possible anti-inflammatory mechanisms of AC5, for example, the activation of immunosuppressive regulatory T cells, production of gut mucosal SIgA antibody, or alteration in gut microbial metabolites (e.g., short-chain fatty acid levels) are required further investigations.

## Data Availability Statement

This work was supported by the Faculty of Medicine Research Fund (MIC-2561-05411), Chiang Mai University, Chiang Mai, Thailand, and the Thailand Research Fund (MRG-6180187) (PT).

## Ethics Statement

The animal study was reviewed and approved by the Animal Care and Use Committee, Chiang Mai University, Thailand in accordance with the Association for Assessment and Accreditation of Laboratory Animal Care (AAALAC) guidelines (Approval No. 2559/MC-0005).

## Author Contributions

PT generated the research. PT and SB designed and conceptualized the research. SB, CS, MN, and PT performed the research. SN provided the probiotic *L. reuteri* KUB AC5 and supervised the study. TK performed the histopathological study. SB, MN, and PT analyzed the data. PT, SB, KN, and MN wrote and reviewed the manuscript. All authors contributed to the article and approved the submitted version.

## Conflict of Interest

The authors declare that the research was conducted in the absence of any commercial or financial relationships that could be construed as a potential conflict of interest.

## Publisher’s Note

All claims expressed in this article are solely those of the authors and do not necessarily represent those of their affiliated organizations, or those of the publisher, the editors and the reviewers. Any product that may be evaluated in this article, or claim that may be made by its manufacturer, is not guaranteed or endorsed by the publisher.
