# Supplementary material for: Anti-inflammatory Effect of Probiotic Limosilactobacillus reuteri KUB-AC5 Against Salmonella Infection in a Mouse Colitis Model
Source: Front Microbiol. 2021 Aug 23;12:716761. doi: 10.3389/fmicb.2021.716761 (PMC8419263; doi:10.3389/fmicb.2021.716761)
Supplement: Supplementary file 1 [file Data_Sheet_1.PDF]

Supplementary material (Buddhasiri S, et al.)

Anti-Inflammatory Effect of Probiotic *Limosilactobacillus reuteri* KUB-AC5 Against *Salmonella* Infection in a Mouse Colitis Model

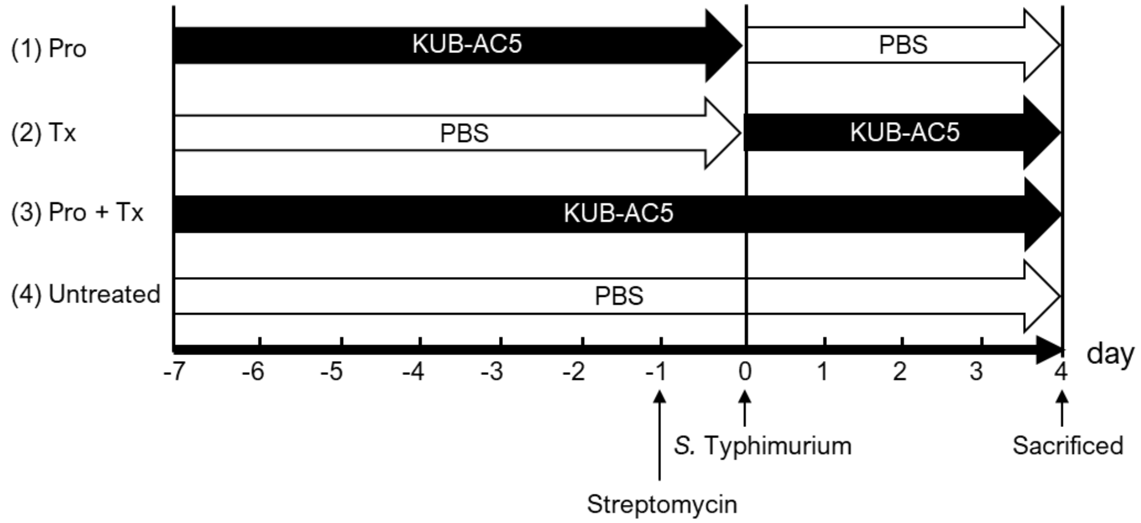

Figure S1 Schematic illustration of the study design. Female 6-8 week-old C57BL/6 mice were divided into four groups (6-7 mice per group). The first group of mice [(1), prophylactic group, Pro] were orally fed with 10^9 cfu AC5 daily for 7 days before STM infection. The second group of mice [(2), therapeutic group, Tx] were fed with 10^9 cfu AC5 daily for 4 days after STM infection. The third group of mice [(3), combined group, Pro + Tx] were continuously fed with 10^9 cfu AC5 daily for 11 days until sacrificed. The fourth group of mice [(4), untreated group] were fed with sterile PBS. 100 µL of (200 mg/mL) streptomycin sulfate was orally fed to mice one day prior to 10^9 cfu STM IR715 infection.

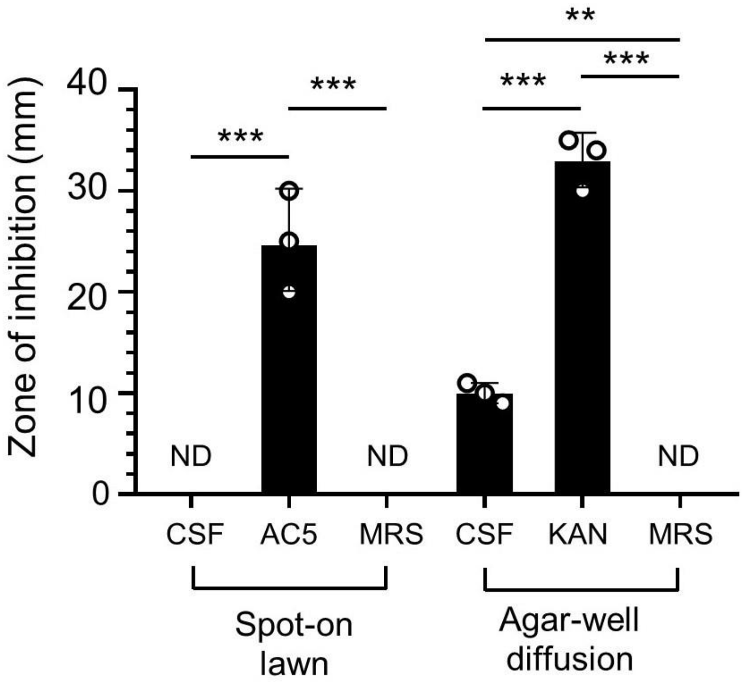

Figure S2 Diameter of inhibition zone in the spot-on lawn assay and agar well diffusion assay. CSF; Cell-free supernatants, AC5; *L. reuteri* KUB-AC5, KAN; kanamycin as a positive control, MRS; de Man, Rogosa, and Sharpe as a negative control, ND; non detectable. Bars represent geometric means ± SDs, ***P* <0.01 and ****P* < 0.001 compared to all other groups.

Table S1. Primers used in this study

Organism	Target gene	Sequence	Reference
Mus musculus	*Gapdh*	5'-TGTAGACCATGTAGTTGAGGTCA-3'<br>5'-AGGTCGGTGTGAACGGATTTG-3'	(1)
	Kc	5'-TGCACCCAAACCGAAGTCAT-3'<br>5'-TTGTCAGAAGCCAGCGTTCAC-3'	
	Nos2	5'-CCAGCCTTGCATCCTCATTGG-3'<br>5'-CCAAACACCAAGCTCATGCGG-3'	
	Ifng	5'- TCAAGTGGCATAGATGTGGAAGAA-3'<br>5'- TGGCTCTGCAGGATTTTCATG-3'	(2)
	Il-6	5'-GCACAACTCTTTTCTCATTTCCACG-3'<br>5'-GCCTTCCCTACTTCACAAGTCCG-3'	(3)
	Zo-1	5'-CAGGGCTCTTTGGAGGAA-3'<br>5'-TACACGATCGTGGCAATAAAC-3'	(4)
L. reuteri KUB-AC5	A hypothetical AC5-specific antimicrobial peptide encoding gene	5'-TCGCTCACGGCTGTTAGGACA-3'<br>5'-AGCACTCCACGTTGCCACA-3'	(5)

Table S2. Cecal histopathology score criteria (6)

Score	Neutrophils infiltration	Mononuclear leukocyte infiltration	Submucosal edema	Epithelial damage	Exudate
0	No changes (0-5)	No changes (0-5)	No changes	No changes	No changes
1	6-20	6-10	Detectable (<10%)	Desquamation	Slight accumulation
2	21-60	11-20	Mild (10-20%)	Mild erosion and mild loss of goblet cells / undifferentiated enterocytes hyperplasia	Mild accumulation
3	61-100	21-40	Moderate (21-40%)	Marked erosion and moderate loss of goblet cells / undifferentiated hyperplasia	Moderate accumulation
4	>100	>40	Marked (>40%)	Ulceration and marked loss of goblet cells / undifferentiated hyperplasia	Marked accumulation

References for the supplementary material

1. Winter SE, Thiennimitr P, Winter MG, Butler BP, Huseby DL, Crawford RW, et al. Gut inflammation provides a respiratory electron acceptor for Salmonella. Nature. 2010;467(7314):426-9.

2. Winter SE, Thiennimitr P, Nuccio SP, Haneda T, Winter MG, Wilson RP, et al. Contribution of Flagellin Pattern Recognition to Intestinal Inflammation during Salmonella enterica Serotype Typhimurium Infection. Infection and immunity. 2009;77(5):1904-16.

3. Xavier MN, Winter MG, Spees AM, Nguyen K, Atluri VL, Silva TM, et al. CD4+ T cell-derived IL-10 promotes Brucella abortus persistence via modulation of macrophage function. PLoS pathogens. 2013;9(6):e1003454.

4. Inagaki-Ohara K, Sawaguchi A, Suganuma T, Matsuzaki G, Nawa Y. Intraepithelial lymphocytes express junctional molecules in murine small intestine. Biochem Biophys Res Commun. 2005;331(4):977-83.

5. Sobanbua S, Dolkittikul S, Nakphaichit M, Keawsompong S, Nitisinprasert S. Antimicrobial peptide presenting potential strain-specific real time polymerase chain reaction assay for detecting the probiotic Lactobacillus reuteri KUB-AC5 in chicken intestine. Poult Sci. 2019.

6. Thiennimitr P, Winter SE, Winter MG, Xavier MN, Tolstikov V, Huseby DL, et al. Intestinal inflammation allows Salmonella to use ethanolamine to compete with the microbiota. Proceedings of the National Academy of Sciences of the United States of America. 2011;108(42):17480-5.
